# Effects of short duration stress management training on self-perceived depression, anxiety and stress in male automotive assembly workers: a quasi-experimental study

**DOI:** 10.1186/1745-6673-3-28

**Published:** 2008-11-21

**Authors:** BA Edimansyah, BN Rusli, L Naing

**Affiliations:** 1 School of Dental Sciences, Universiti Sains Malaysia Health Campus, 16150 Kubang Kerian, Kelantan, Malaysia; 2 Clinical School Johor Bahru, Tan Sri Jeffrey Cheah School of Medicine, Monash University, JKR 1235, Bukit Azah, 80100 Johor Bahru, Johor, Malaysia; 3 Institute of Medicine, National University of Brunei Darussalam, Jalan Tungku Link, Gadong BE 1410, Brunei Darussalam

## Abstract

To examine the effects of short duration stress management training (SMT) on self-perceived depression, anxiety and stress in male automotive assembly workers, 118 male automotive workers from Pekan, Pahang (n = 60, mean age = 40.0 years, SD = 6.67) and Kota Bharu, Kelantan (n = 58, mean age = 38.1 years, SD = 5.86) were assigned to experimental and control group, respectively. A SMT program consisting of aerobic exercise, stress management manual, video session, lecture, question and answer session, and pamphlet and poster session were conducted in the experimental group. A validated short-form Malay version of the Depression Anxiety Stress Scales (DASS-21) were self-administered before and after the intervention program in the experimental and control group and their time and group interaction effects were examined using the repeated measure ANOVA test. Results indicated that the mean (SD) scores for DASS-Depression (p = 0.036) and DASS-Anxiety (p = 0.011) were significantly decreased, respectively, after the intervention program in the experimental group as compared to the control group (significant time-group interaction effects). No similar effect was observed for the mean (SD) scores for DASS-Stress (p = 0.104). However, the mean (SD) scores for subscales of DASS-Depression (Dysphoria, p = 0.01), DASS-Anxiety (Subjective Anxiety, p = 0.007, Situational Anxiety, p = 0.048), and DASS-Stress (Nervous Arousal, p = 0.018, Easily Upset, p = 0.047) showed significant time and group interaction effects. These findings suggest that short duration SMT is effective in reducing some aspects of self-perceived depression, anxiety and stress in male automotive workers.

## Background

Job stress is a major occupational health problem in many sectors of industries and automotive assembly industry workers are one of several occupational groups who report disproportionately high levels of job stress [1,2]. Studies have shown that job stress is a significant problem in automotive assembly line workers [2-6]. For instance, Karasek has highlighted high strain work (high demand and low control) among machine-paced operative assemblers [3]. Kvanström reported that automotive assembly-line work is often performed in a workplace environment with physical problems, such as noise, vibrations and dangerous machines that can be important factors of stress. Furthermore, technical development in assembly-line work, especially in large companies, has often resulted in more complicated tasks for the workers who may have difficulty in over-viewing all the steps in production; this can easily build up a fear of the unknown and, consequently, more stress [1]. Considering these problems, it is desirable to implement a stress management intervention in this occupational setting.

Several stress intervention programs have been developed over the years to counter health problems in the workplace. According to van der Klink et al. [7], interventions designed to reduce job stress and its health effects can be categorized according to their focus, content, method, and duration. With regards to focus, intervention can be categorized into two basic approaches: a) individual-focused approach, which aims to increase individual psychological resources and responses such as coping; and b) organization-focused intervention, which aims to improve stressful work environments through organizational development and job redesign. The present study is aimed at the individual-focused intervention.

There are various techniques in the individual-focused approach that include exercise, stress management, relaxation training, physical fitness, cognitive-behavioural training, meditation, biofeedback, hypnosis, yoga, and interpersonal skills. A recent meta-analysis reported that job stress management intervention studies that focus on the individuals are effective in reducing workers' stress-related complaints [7]. Among the stress management interventions in the individual-oriented approach, exercise and stress management are among the strategies that are found to significantly reduce the long term effects of health outcomes [7-14]. However, no study has tested the effects of a short duration, easy-to-implement SMT that could reduce depression, anxiety and stress in an occupational setting such as those in the automotive assembly industry. Therefore, in the present study, we evaluate the effectiveness of a short duration SMT in reducing self-perceived depression, anxiety and stress among male automotive assembly plant workers in Malaysia.

## Methods

### Study design

A quasi experimental study was conducted among male automotive workers in Pahang and Kelantan from July to September, 2006. This study is part of a national research project entitled "Occupational Stress Intervention Study in Petrochemical and Automotive Assembly Plants: Developing and Evaluating a Stress Management Program at Workplaces" (OSIS) under the research program "Quality of Work Life – National Occupational Risk Management Study and National Injury Prevention" (NORMS) funded by the Ministry of Science, Technology and Innovation (MOSTI), Malaysia under its 8^th ^Malaysia Plan.

### Sample size estimation

Sample size was estimated using the PS Software for two sample means, with power of 80% and alpha of 5%. Sample size calculation was based on the standard deviation (SD = 10.12) for self-perceived stress in general practice patients with mental health problems in Australia [15] and the calculated sample size was 49. After considering a 10% non-response, the estimated sample size was 54 automotive workers.

### Recruitment of study subjects

The reference population consists of automotive workers in Malaysia. The source population includes workers in an automotive plant in Pekan, Pahang and workers in an automotive plant in Kota Bharu, Kelantan. The sampling frame is the list of automotive workers available for screening using the DASS questionnaire in Pekan, Pahang (88 workers) and Kota Bharu, Kelantan (75 workers). Inclusion criteria include male automotive workers and at least one year of working experience. Exclusion criterion is a history of medically confirmed mental illnesses. Based on the inclusion and exclusion criteria, 60 automotive workers were eligible in Pekan, Pahang (assigned to the experimental group) and 58 automotive workers in Kota Bharu, Kelantan (assigned to the control group).

### Stress management training

Our intervention program consists of a short duration SMT module that includes the following sessions: aerobic exercise, pamphlet and poster session on stress reduction through adopting a healthy lifestyle, review session of a simplified and well illustrated manual on the prevention and control of stress in the workplace, interactive lecture session on stress management in the workplace, video session on stress management in the workplace, and question and answer session. The intervention program was held in a public facility adjacent to the automotive plant in Pekan, Pahang.

#### Session 1

Aerobic Exercise. The aerobic exercise session was conducted by qualified aerobic instructors and consists of a preparatory 15 minutes warming up stretching exercises [13]. This was followed by low intensity walking and jogging routines [16]. Participants were then taken through a series of rhythmic dancing and running routines that lasted about 30 minutes. In the last 15 minutes, participants carried out a cooling down manoeuvre that includes deep breathing exercises and relaxation of over-stretched muscles [16].

### Session 2

Pamphlet and Poster Session. This is an interactive pamphlet and poster session where participants were guided by the researchers to the pamphlets and posters that depict the nature, causes, and signs and symptoms of stress, stress reduction through adopting a healthy life style, and methods of managing stress in the workplace. A short question and answer session was conducted to improve the participants' understanding of the materials presented.

#### Session 3

Manual Review Session. A simplified and well illustrated manual on the prevention and control of stress in the workplace was distributed to all participants and they were asked to review it over the next 30 minutes followed by a question and answer session.

#### Session 4

Lecture on Stress Management. This is an interactive lecture session on stress management in the workplace and designed to reinforce the participants' understanding of occupational stress and a suggested plan towards stress reduction and control. The key component of the lecture is an elaboration of occupational stress, risk factors involved, associated health impacts and ways to prevent and control stress in the workplace. References were made to the Job Demand Control Model of stress and how work conditions of high job demand, low job control and poor social support can result in severe stress and serious health consequences. Participants were carefully guided as to the various ways of dealing with stress at work and the importance of good social support in protecting workers from severe stress.

#### Session 5

Video Session on Stress Management in the Workplace. The video session is entitled "Stress Management in the Malaysian Automotive Industry" and was produced by the researchers as part of the research program, with permission and full cooperation from the automotive assembly plant. The video previewed all processes of production in the automotive industry. The aim of this session is to provide an overview of the working conditions in the automotive assembly plant and how the working conditions can contribute toward occupational stress. The video also showed several simple methods of dealing with stress in the workplace.

#### Session 6

Question and Answer Session. At the end of the video session, the researchers conducted a question and answer session with the participants. This session was aimed at exploring several practical problems related to occupational stress encountered by the participants and discussing several options and suggestions in managing these problems. Participants were asked to identify several stressful situations that they had personally experienced at work and how they cope with these stresses. Suggestions were made to strengthen their coping skills including the need to adopt the healthy lifestyle approach – regular exercise, avoidance of tobacco, drugs, and alcohol, adequate sleep and rest, and proper nutrition and dieting – that has been shown to be beneficial in stress reduction.

### Validated short-form Malay version of the depression anxiety stress scales (DASS-21)

DASS-21 is the short-form of Lovibond and Lovibond's 42-item self-report which measures the negative emotional states of depression, anxiety and stress [17]. The questionnaire consists of three scales: DASS-Depression Scale (Cronbach's alpha = 0.81), DASS-Anxiety Scale (Cronbach's alpha = 0.85) and DASS-Stress Scale (Cronbach's alpha = 0.85) [18]. The DASS-Depression Scale assesses dysphoria, hopelessness, devaluation of life, self-deprecation, lack of interest/involvement, anhedonia and subjective experience of anxious affect. The DASS-Anxiety scale assesses autonomic arousal, skeletal muscle effects, situational anxiety, and subjective experience of anxious affect. The DASS-Stress scale is sensitive to levels of chronic non-specific arousal. It assesses difficulty relaxing, nervous arousal, and being easily upset/agitated, irritable/over reactive, and impatient. Subjects are asked to use a 4-point severity/frequency scale (0 = Did not apply to me at all, 1 = Applied to me to some degree, or some of the time, 2 = Applied to me a considerable degree, or a good part of the time, and 3 = Applied to me very much, or most of the time) to rate the extent to which they have experienced each state over the past week. Scores for DASS-Depression, DASS-Anxiety and DASS-Stress were calculated by summing the scores for the relevant items, multiplying by 2 and converting these scores into percentile scores [17]. The DASS scale manual [17] provides a series of cut-off values to classify individuals into 5 levels of severity (normal, mild, moderate, severe, and extremely severe). Based on the cut-off percentiles, workers scoring less than 78 percentiles are considered normal; 78 to 87 percentiles as mild; 85 to 95 percentiles as moderate; 95–98 percentiles as severe; and 98–100 percentiles as extremely severe.

To examine the effect of short duration SMT intervention program on self-perceived depression, anxiety and stress, the DASS-21 questionnaires were self-administered to both study groups before and after the intervention program.

### Statistical analysis

Data entry and analysis was done using SPSS version 12.0.1 (SPSS Inc, Chicago, IL, USA). Mean and standard deviations (SD) were calculated for continuous variables, and frequencies and percentages for categorical variables. Effectiveness of the short duration SMT program was examined using the repeated measure ANOVA analysis where the time-group interaction effect (within subject-between subject) was modelled. Assumptions of the repeated measure ANOVA include the following: histogram of residuals for normality assumption and Levene's test for equal variance assumption; sphericity assumption is not necessary because the model includes only 2 repeated measures; model fitness will be checked by residual plots and lack-of-fit test; level of significance is set at α = 0.05.

## Results

### Socio-demographic characteristics

A total of 118 workers (60 workers in experimental group and 58 workers in control group) participated in this study. Socio-demographic characteristics such as age, marital status, years of education, duration of work and salary were compared between the two groups (Table 1). Except for marital status, all socio-demographic characteristics were found to be not significantly different between the two study groups.

**Table 1:** Socio-demographic characteristics of experimental group (n = 60) and control group (n = 58)

Socio-demographic characteristics	Experimental group (*n *= 60)	Control group (*n *= 58)	*t*-statistic (*df*)	*X*^2 ^(*df*)	*p*-value
				
	Mean (SD)	Mean (SD)			
Age (year)	40.0 (6.67)	38.1 (5.86)	1.580 (*116*)		0.117
Salary (RM)	2952.7 (2048.14)	2645.46 (2058.34)	0.782 (*108*)		0.436
Duration of work (year)	12.4 (10.60)	5.6 (5.26)	1.795 (*115*)		0.075
Education (year)	14.0 (2.31)	14.1 (2.09)	-0.339 (*116*)		0.735
Marital status	No. (%)	No. (%)			
a. Single	3 (5.0)	42 (72.4)		56.8 (*1*)	<0.001
b. Married	57 (95.0)	16 (27.6)			

### Effects of short duration SMT on mean (sd) scores for DASS-depression, DASS-anxiety and DASS-stress scales

**Figure 1:**
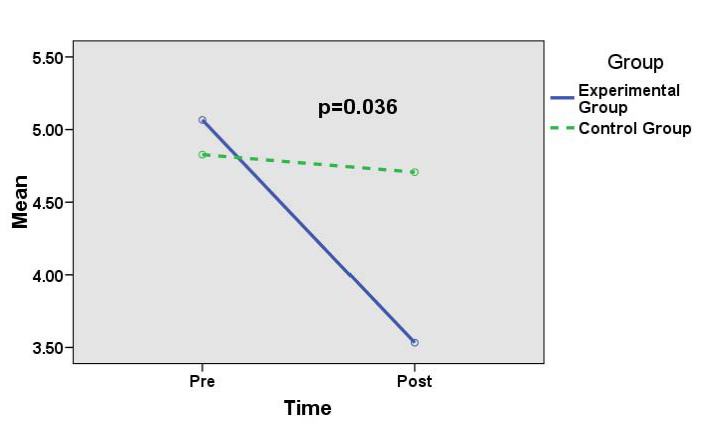
**Comparing mean (sd) scores for DASS-depression between experimental group (n = 60) and control group (n = 58) (repeated measure anova)**.

**Figure 2:**
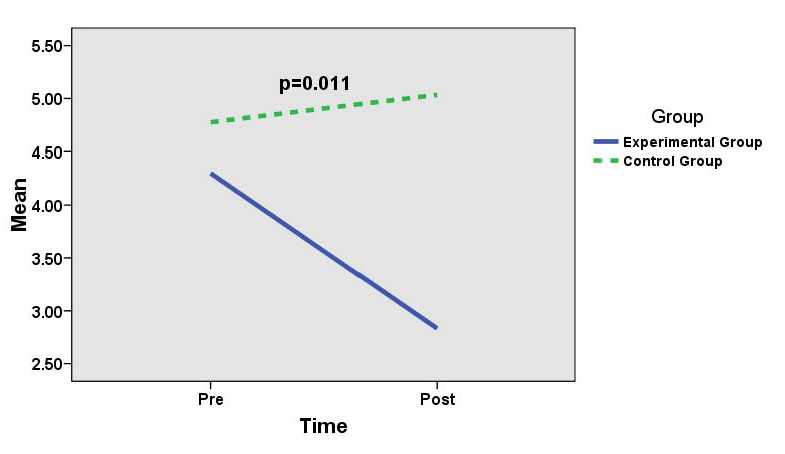
**Comparing mean (sd) scores for DASS-anxiety between experimental group (n = 60) and control group (n = 58) (repeated measure anova)**.

**Figure 3:**
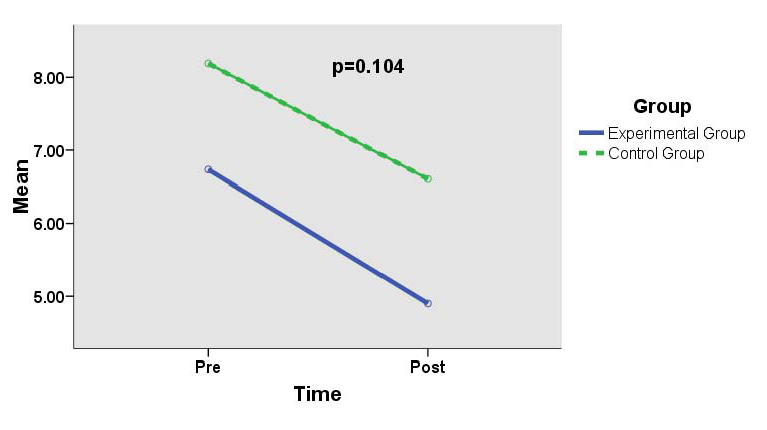
**Comparing mean (sd) scores for DASS-stress between experimental group (n = 60) and control group (n = 58) (repeated measure anova)**.

Table 2 and Figures 1, 2 and 3 show the effects of SMT on the mean (SD) scores (pre- and post-intervention) for DASS-Depression, DASS-Anxiety and DASS-Stress scales in the experimental group as compared to those in the control group. There are significantly reduced mean (SD) scores for DASS-Depression (*p *= 0.036) and DASS-Anxiety (*p *= 0.011) in the experimental group as compared to those in the control group (significant time-group interaction effects). However, there is no significant difference in the mean (SD) scores (pre- and post-intervention) for DASS-Stress (*p *= 0.104) in the experimental group as compared to those in the control group (not significant time-group interaction effect).

**Table 2:** Comparing mean (sd) scores for DASS-depression, DASS-anxiety and DASS-stress between experimental group (n = 60) and control group (n = 58) (repeated measure anova)

Variables	Time	Experimental group *(n *= 60)	Control group (*n *= 58)	*F *stat. (*df*)^a^	*p *value
	
		Mean (SD)	Mean (SD)		
DASS-Depression	Pre	5.1 (3.07)	4.8 (3.43)	6.15 (1;116)	0.036
	Post	3.5 (2.88)	4.7 (3.69)		
DASS-Anxiety	Pre	4.3 (3.17)	4.8 (3.49)	6.621(1;116)	0.011
	Post	2.8 (2.77)	5.0 (2.95)		
DASS-Stress	Pre	6.7 (3.35)	8.2 (3.62)	2.686 (1;116)	0.104
	Post	5.5 (2.80)	8.1 (3.47)		

^a ^time-group interaction effect

### Effects of short duration SMT on mean (sd) scores for DASS-depression, DASS-anxiety and DASS-stress subscales

Table 3 shows the effects of SMT on the mean (SD) scores (pre- and post-intervention) for DASS-Depression, DASS-Anxiety and DASS-Stress subscales in the experimental group as compared to those in the control group. For DASS-Depression Subscales, there is a significant time-group interaction effect in the mean (SD) scores for dysphoria (*p *= 0.010) but not for evaluation of life (*p *= 0.200), hopelessness (*p *= 0.370), self-deprecation (*p *= 0.076), anhedonia (*p *= 0.352) and inertia (*p *= 0.350). Meanwhile, for DASS-Anxiety Subscales, there are significant time-group interaction effects in the mean (SD) scores for anxiety (*p *= 0.007) and situational anxiety (*p *= 0.048), but not for skeletal muscle effect (*p *= 0.495) and autonomic arousal (0.054). Whereas for DASS-Stress Subscales, there are significant time-group interaction effects in the mean (SD) scores for nervous arousal (*p *= 0.018) and easily upset (*p *= 0.047) but not for difficulty relaxing (*p *= 0.750) and irritable (*p *= 0.722).

**Table 3:** Comparing mean (sd) scores for DASS-depression, DASS-anxiety and DASS-stress subscales between experimental group (n = 60) and control group (n = 58) (repeated measure anova)

		Experimental group *n *= 60	Control group *n *= 58		
				
DASS-depression subscales	Time	Mean (SD)	Mean (SD)	*F *stat. (*df*)^a^	*p *value
Dysphoria	Pre	1.0 (0.59)	0.7 (0.78)	6.810 (1;116)	0.010
	Post	0.6 (0.61)	0.8 (0.83)		
Devaluation of Life	Pre	0.8 (1.06)	0.9 (1.14)	1.659 (1;116)	0.200
	Post	0.6 (0.82)	1.0 (1.27)		
Hopelessness	Pre	0.6 (0.62)	0.8 (0.92)	0.811 (1;116)	0.370
	Post	0.4 (0.52)	0.7 (0.87)		
Self Deprecation	Pre	0.8 (0.70)	0.8 (0.81)	3.201 (1;116)	0.076
	Post	0.6 (0.62)	0.8 (0.80)		
Anhedonia	Pre	0.8 (0.77)	0.6 (0.73)	0.874 (1;116)	0.352
	Post	0.6 (0.76)	0.6 (0.77)		
Inertia	Pre	1.1 (0.71)	1.0 (0.79)	0.882 (1;116)	0.350
	Post	0.9 (0.65)	0.8 (0.87)		

DASS-anxiety subscales					

Subjective Anxiety	Pre	0.7 (0.89)	0.7 (0.12)	7.058 (1;116)	0.007
	Post	0.5 (0.77)	1.2 (1.30)		
Situational Anxiety	Pre	1.0 (0.76)	1.2 (1.02)	4.009 (1;116)	0.048
	Post	0.8 (0.62)	1.3 (0.96)		
Skeletal Muscle Effect	Pre	0.5 (0.68)	0.5 (0.82)	0.469 (1;116)	0.495
	Post	0.3 (0.54)	0.5 (0.68)		
Autonomic Arousal	Pre	2.2 (1.58)	2.4 (1.68)	3.791 (1;116)	0.054
	Post	1.3 (1.43)	2.1 (1.64)		

DASS-stress subscales					

Difficulty Relaxing	Pre	1.9 (1.14)	1.8 (1.38)	0.102 (1;116)	0.750
	Post	1.6 (0.72)	1.5 (1.22)		
Nervous Arousal	Pre	0.9 (0.77)	1.0 (0.90)	5.778 (1;116)	0.018
	Post	0.6 (0.59)	1.1 (0.81)		
Easily Upset	Pre	1.0 (0.68)	1.6 (0.81)	4.042 (1;116)	0.047
	Post	0.9 (0.70)	1.1 (0.97)		
Irritable	Pre	1.9 (1.16)	2.3 (1.41)	0.127 (1;116)	0.722
	Post	1.4 (1.03)	1.9 (1.51)		

^a ^time and group interaction effect

## Discussion

We examined the effects of short duration SMT on self-perceived depression, anxiety and stress in automotive workers using the validated short-form Malay version of the Depression Anxiety Stress Scales (DASS-21). Results indicated that SMT is effective in reducing some aspects of self-perceived depression, anxiety, and stress in these workers.

Blumenthal et al [13] have shown that combined exercise for 35 minutes 3 times per week for 16 weeks plus weekly 1.5-hour SMT for 16 weeks was effective in reducing general distress as measured by the General Health Questionnaire (*p *= 0.02), depressive symptoms as measured by the Beck Depression Inventory (*p *= 0.02) and improved markers of cardiovascular risk more than usual medical care alone. Our present study also shows that short duration SMT is effective in reducing some aspects of self-perceived depression, anxiety and stress in automotive workers. These findings are very important because automotive assembly workers tend to be busy, and long duration of training would be less likely to be used. However, it should be noted that our study did not consider compliance or continuation of the present training. Further research must be carried out to explore how the subjects would respond to longer duration training of weeks or even months.

To our knowledge, there is only one empirical study that examined the immediate effects of an intervention program [19]. Taniguchi et al [19] have shown the immediate effects of a stress management program that combines lecture (1 hour) and relaxation training (10 minutes) on Japanese female medical co-workers indicated that salivary immunoglobulin A (s-IgA) levels in the relaxation group increased after relaxation training compared to those in the control group (*p *= 0.03). However, no significant relaxation training effects were found in the Profile Mood States (POMS) subscales for tension-anxiety (*p *= 0.870), depression-dejection (*p *= 0.161), anger-hostility (*p *= 0.937), vigor-activity (*p *= 0.657) and fatigue-inertia (*p *= 0.212), except for confusion (*p *= 0.036). Since the scope of the present study is only focused on responses using self-perceived DASS21 questionnaire, we suggest that further studies using physiological responses are needed to explore the effectiveness of SMT.

Studies that examine the effects of short duration SMT on stressful workers are seriously lacking. One reason for this lack is that senior managers in the same organization presume that formal exercise training and SMT programs are time-consuming, expensive and workers cannot spare the time to practice any coping skills. Another reason is that provided by Yung *et al*. [20], with given examples in hospital setting, that there is the belief that such intervention programs usually require manpower and resources that could be better spent on other patient care than on staff care. However, given the high stress levels reported among automotive workers in previous studies, including those in Malaysia, these reasons are not sufficient to ignore the well-being of automotive assembly workers. Therefore, short duration intervention programs such as SMT are needed for automotive workers in Malaysia.

In this intervention program, the experimental group received a short duration SMT program (aerobic exercise, manual, video session, lecture, pamphlet and poster sessions) that shows improvement in the mean (SD) scores for DASS-Depression and DASS-Anxiety but not for DASS-Stress. However, when we examined the mean (SD) scores for DASS-Stress (pre and post-intervention), the mean (SD) scores were slightly reduced after the intervention program. In an effort to identify which subscales of DASS-Depression, DASS-Anxiety and DASS-Stress are significantly contributing towards improvement in the mean (SD) scores, we also analysed the effects of the intervention program on each subscale of the DASS-Depression, DASS-Anxiety and DASS-Stress scales. With regards to DASS-Depression subscales, we found that the mean (SD) score for dysphoria (*p *= 0.010) was significantly reduced after intervention. This could be due to the effect of the SMT intervention program, especially the aerobic exercise. However, the mean (SD) scores (pre- and post-intervention) for DASS-Depression subscales such as devaluation of life (*p *= 0.200), hopelessness (*p *= 0.370), self deprecation (*p *= 0.076), anhedonia (*p *= 0.352) and inertia (*p *= 0.350) were not significantly different in the experimental group as compared to those in the control group. All mean (SD) scores for DASS-Depression subscales were slightly reduced after the intervention program.

With regards to the mean (SD) scores for DASS-Anxiety subscales, we found that the mean (SD) scores for subjective anxiety (*p *= 0.007) and situational anxiety (*p *= 0.048) were significantly reduced after intervention. This suggests that subjective anxiety, as explained by "I had a feeling of faintness" and "I felt scared without any good reason" and situational anxiety as explained by "I found myself in situations that made me so anxious I was most relieved when they ended" are statistically significantly reduced. Meanwhile, the mean (SD) scores for self-perceived DASS-Anxiety subscales, such as skeletal muscle effect (*p *= 0.495) and autonomic arousal (*p *= 0.054) were not significantly different pre- and post-intervention. However, when we examined the mean (SD) scores for pre- and post-intervention, all the mean (SD) scores for DASS-Anxiety subscales were also reduced.

Whereas for DASS-Stress subscales, we found that the mean (SD) scores for nervous arousal ("I felt that I was using a lot of nervous energy") (*p *= 0.018) and easily upset ("I found myself getting upset by quite trivial things and I found myself getting upset rather easily") (*p *= 0.047) were significantly reduced after intervention.

In this study, we suggest that short duration SMT intervention program, combining aerobic exercise, manual, video session, lecture, pamphlet and poster presentation, and question and answer session, could be used in automotive industries as part of their social and welfare program and implemented as a weekend or monthly activity for the workers.

### Limitations of the study

Several limitations of this study should be noted. Firstly, in the aerobic exercise training, participants were trained by instructors only once. The exercise training of approximately one hour may have been too short. Therefore, it is necessary to encourage the participants towards regular practice. Because self-perceived depression, anxiety and stress were measured before and after the short duration SMT program, therefore, the findings related to the short-term effects could not be compared with previous studies that were mostly related to long-term effects. The present study could not provide data on how long the effects remain after the intervention program, or on any cumulative effects of the intervention program through SMT program.

## Conclusion

Reducing depression, anxiety and stress in automotive assembly workers by stress intervention program is an important issue. The present study was conducted to examine the effects of short duration SMT on self-perceived depression, anxiety and stress in automotive workers using the validated short-form Malay version of the Depression Anxiety Stress Scales (DASS-21). Results indicated that SMT is effective in reducing some aspects of self-perceived depression, anxiety, and stress in these workers. Therefore, intervention programs to reduce stress, anxiety, and depression in the workplace may facilitate automotive workers to provide high-quality service in an automotive assembly plant setting.

## Competing interests

The authors declare that they have no competing interests.

## Authors' contributions

RBN, EBA and LN contributed equally to the design and conduct of the survey, analysis of the results, drafting and critical revision of the manuscript. RBN, EBA and LN read and approved the final version of the manuscript

## References

[B1] KvarnstrÖm S (1997). Stress prevention for blue-collar workers in assembly-line production.

[B2] Oleske DM, Neelakantan J, Andersson GB, Hinrichs BG, Lavender SA, Morrissey MJ, Zold-Kilbourn P, Taylor E (2004). Factors affecting recovery from work-related, low back disorders in autoworkers. Arch Phys Med Rehabil.

[B3] Karasek R, Baker D, Marxer F, Ahlbom A, Theorell T (1981). Job decision latitude, job demands, and cardiovascular disease: a prospective study of Swedish men. Am J Public Health.

[B4] Kumlin L, Latscha G, Orth-Gomer K, Dimberg L, Lanoiselee C, Simon A, Eriksson B (2001). Marital status and cardiovascular risk in French and Swedish automotive industry workers – cross sectional results from the Renault-Volvo Coeur study. J Intern Med.

[B5] Hanse JJ, Forsman M (2001). Identification and analysis of unsatisfactory psychosocial work situations: a participatory approach employing video-computer interaction. Appl Ergon.

[B6] Lottridge D (2004). Work at the Uddevalla Volvo Plant from the perspective of the demand-control model. Bull Sci Tech Society.

[B7] Klink JJ van der, Blonk RW, Schene AH, van Dijk FJ (2001). The benefits of interventions for work-related stress. Am J Public Health.

[B8] Shimazu A, Okada Y, Sakamoto M, Miura M (2003). Effects of stress management program for teachers in Japan: A pilot study. J Occup Health.

[B9] Rhenen WV, Roland WB, Blonk Jac JL, Klink JJ van der, Frank JH, van Dijk Wilmar B (2005). The effect of a cognitive and a physical stress-reducing programme on psychological complaints. Int Arch Occup Environ Health.

[B10] Kagan N, Kagan H, Watson MG (1995). Stress reduction in the workplace: The effectiveness of psycholoedutional programs. J Counseling Psycho.

[B11] Reynolds S, Shapiro D (1991). Stress reduction in transition: Conceptual problems in the design, implementation and evaluation of worksite stress management intervention. Human Relations.

[B12] Selkowitz D, Kulig K, Poppert E, Flanagan S, Matthews N, Beneck G, Popovich J, Lona J, Yamada K, Burke W, Ervin C, Powers C, Physical Theraphy Clinical Research Network (PTClinResNet) (2006). The immediate and long-term effects of exercise and patient education on physical, functional, and quality-of-life outcome measures after single-level lumbar microdiscectomy: a randomized controlled trial protocol. BMC Musculoskeletal Disorders.

[B13] Blumenthal J, Sherwood A, Babyak M, Watkins L, Waugh R, Georgiades A, Bacon S, Hayano J, Coleman R, Hinderliter A (2005). Effects of exercise and stress management training on markers of cardiovascular risk in patients with ischemic heart disease: A randomized controlled trial. JAMA.

[B14] Blumenthal J, Sherwood A, Babyak M, Watkins L, Waugh R, Georgiades A, Bacon S, Hayano J, Coleman R, Hinderliter A (1997). Stress management and exercise training in cardiac patients with myocardial ischemia: effects on prognosis and evaluation of mechanisms. Arch Intern Med.

[B15] Vines R, Richards J, Thomson D, Brechman-Toussaint M, Kluin M, Vesely L (2004). Clinical psychology in general practice: a cohort study. Med J Aust.

[B16] Seaward BK (2004). Managing stress:principles and strategies for health and well-being.

[B17] Lovibond SH, Lovibond PF (2002). Manual for the Depression Anxiety Stress Scales. 2 edition.

[B18] Mazalisah M, Rusli B, Naing L, Edimansyah B (2005). Validation of the Malay version of the Depression Anxiety Stress Scales 21-item in an automobile industry. Malaysian J Med Sci.

[B19] Taniguchi T, Hirokawa K, Tsuchiya M, Kawakami N (2007). The immediate effects of 10-minute relaxation training on salivary immunoglobulin A (s-IgA) and mood state for Japanese female medical co-workers. Acta Med Okayama.

[B20] Yung P, Fung M, Chan T, Lau B (2004). Relaxation training methods for nurse managers in Hong Kong: a controlled study. Int J Ment Health Nurs.

